# Genetic Contribution to Alcohol Dependence: Investigation of a Heterogeneous German Sample of Individuals with Alcohol Dependence, Chronic Alcoholic Pancreatitis, and Alcohol-Related Cirrhosis

**DOI:** 10.3390/genes8070183

**Published:** 2017-07-17

**Authors:** Jens Treutlein, Josef Frank, Fabian Streit, Céline S. Reinbold, Dilafruz Juraeva, Franziska Degenhardt, Liz Rietschel, Stephanie H. Witt, Andreas J. Forstner, Monika Ridinger, Jana Strohmaier, Norbert Wodarz, Helene Dukal, Jerome C. Foo, Per Hoffmann, Stefan Herms, Stefanie Heilmann-Heimbach, Michael Soyka, Wolfgang Maier, Wolfgang Gaebel, Norbert Dahmen, Norbert Scherbaum, Bertram Müller-Myhsok, Susanne Lucae, Marcus Ising, Felix Stickel, Thomas Berg, Ulla Roggenbuck, Karl-Heinz Jöckel, Henrike Scholz, Ulrich S. Zimmermann, Stephan Buch, Wolfgang H. Sommer, Rainer Spanagel, Benedikt Brors, Sven Cichon, Karl Mann, Falk Kiefer, Jochen Hampe, Jonas Rosendahl, Markus M. Nöthen, Marcella Rietschel

**Affiliations:** 1 Department of Genetic Epidemiology in Psychiatry, Central Institute of Mental Health, Medical Faculty Mannheim, Heidelberg University, 68159 Mannheim, Germany; jens.treutlein@zi-mannheim.de (J.T.); josef.frank@zi-mannheim.de (J.F.); fabian.streit@zi-mannheim.de (F.S.); stephanie.witt@zi-mannheim.de (S.H.W.); jana.strohmaier@zi-mannheim.de (J.S.); helene.dukal@zi-mannheim.de (H.D.); jerome.foo@zi-mannheim.de (J.C.F.); 2 Human Genomics Research Group, Department of Biomedicine, University and University Hospital Basel, 4031 Basel, Switzerland; celine.reinbold@unibas.ch (C.S.R.); forstner@uni-bonn.de (A.J.F.); per.hoffmann@unibas.ch (P.H.); stefan.herms@unibas.ch (S.H.); sven.cichon@unibas.ch (S.C.); 3 Institute of Medical Genetics and Pathology, University Hospital Basel, 4031 Basel, Switzerland; 4 Division of Applied Bioinformatics (G200), German Cancer Research Center (DKFZ), 69120 Heidelberg, Germany; juraeva@yahoo.com (D.J.); b.brors@dkfz.de (B.B.); 5 Institute of Human Genetics, University of Bonn, 53127 Bonn, Germany; franziska.degenhardt@uni-bonn.de (F.D.); sheilman@uni-bonn.de (S.H.-H.); markus.noethen@uni-bonn.de (M.M.N.); 6 Department of Genomics, Life & Brain Center, University of Bonn, 53127 Bonn, Germany; 7 University Hospital of Child and Adolescent Psychiatry and Psychotherapy, University of Bern, 3000 Bern 60, Switzerland; liz.rietschel@kjp.unibe.ch; 8 Department of Psychiatry (U.P.K.), University of Basel, CH-4002 Basel, Switzerland; 9 Department of Psychiatry and Psychotherapy, University of Regensburg, 93053 Regensburg, Germany; monika.ridinger@gmx.ch (M.R.); norbert.wodarz@medbo.de (N.W.); 10 Psychiatric Health Care Aargau, 5210 Windisch, Switzerland; 11 Private Hospital Meiringen, 3860 Meiringen, Switzerland; Michael.Soyka@privatklinik-meiringen.ch; 12 Psychiatric Hospital, Ludwig Maximilians University, 80336 Munich, Germany; 13 Department of Psychiatry, University of Bonn, 53105 Bonn, Germany; Wolfgang.Maier@ukb.uni-bonn.de; 14 Department of Psychiatry and Psychotherapy, University of Düsseldorf, 40629 Düsseldorf, Germany; wolfgang.gaebel@uni-duesseldorf.de; 15 Department of Psychiatry, University of Mainz, 55131 Mainz, Germany; n.dahmen@oehk.de; 16 LVR-Hospital Essen, Department of Psychiatry and Psychotherapy, Medical Faculty, University of Duisburg-Essen, 45147 Essen, Germany; norbert.scherbaum@uni-due.de; 17 Department of Statistical Genetics, Max-Planck-Institute of Psychiatry, 80804 Munich, Germany; bmm@mpipsykl.mpg.de; 18 Department of Psychiatric Pharmacogenetics, Max-Planck-Institute of Psychiatry, 80804 Munich, Germany; lucae@psych.mpg.de; 19 Department of Molecular Psychology, Max-Planck-Institute of Psychiatry, 80804 Munich, Germany; ising@psych.mpg.de; 20 Department of Gastroenterology and Hepatology, University Hospital of Zurich, 8091 Zurich, Switzerland; Felix.stickel@uzh.ch; 21 Hirslanden Private Hospital, 8032 Hirslanden Zürich, Switzerland; 22 Section of Hepatology, University Hospital Leipzig, 04103 Leipzig, Germany; Thomas.Berg@medizin.uni-leipzig.de; 23 Institute for Medical Informatics, Biometry, and Epidemiology, University Hospital of Essen, 45122 Essen, Germany; Ulla.Roggenbuck@uk-essen.de (U.R.); k-h.joeckel@uk-essen.de (K-.H.J.); 24 Department of Animal Physiology, University of Cologne, 50674 Cologne, Germany; henrike.scholz@uni-koeln.de; 25 Department of Psychiatry and Psychotherapy, University Hospital Carl Gustav Carus, Dresden Technical University, 01307 Dresden, Germany; UlrichS.Zimmermann@uniklinikum-dresden.de; 26 Medical Department 1, University Hospital Dresden, TU Dresden, 01307 Dresden, Germany; Stephan.Buch@uniklinikum-dresden.de (S.B.); Jochen.Hampe@uniklinikum-dresden.de (J.H.); 27 Institute of Psychopharmacology, Central Institute of Mental Health, Medical Faculty Mannheim, Heidelberg University, 68159 Mannheim, Germany; wolfgang.sommer@zi-mannheim.de (W.H.S.); rainer.spanagel@zi-mannheim.de (R.S.); 28 Department of Addictive Behavior and Addiction Medicine, Central Institute of Mental Health, Medical Faculty Mannheim, Heidelberg University, 68159 Mannheim, Germany; karl.mann@zi-mannheim.de (K.M.); falk.kiefer@zi-mannheim.de (F.K.); 29 Department of Internal Medicine I, Martin Luther University Halle, 06120 Halle (Saale), Germany; Jonas.Rosendahl@medizin.uni-leipzig.de

**Keywords:** alcohol dependence, chronic alcoholic pancreatitis, alcoholic liver cirrhosis, genome-wide association study, alcohol dehydrogenase, *ADH1B*, *ADH1C*

## Abstract

The present study investigated the genetic contribution to alcohol dependence (AD) using genome-wide association data from three German samples. These comprised patients with: (i) AD; (ii) chronic alcoholic pancreatitis (ACP); and (iii) alcohol-related liver cirrhosis (ALC). Single marker, gene-based, and pathway analyses were conducted. A significant association was detected for the *ADH1B* locus in a gene-based approach (*p*_uncorrected_ = 1.2 × 10^−6^; *p*_corrected_ = 0.020). This was driven by the AD subsample. No association with *ADH1B* was found in the combined ACP + ALC sample. On first inspection, this seems surprising, since *ADH1B* is a robustly replicated risk gene for AD and may therefore be expected to be associated also with subgroups of AD patients. The negative finding in the ACP + ALC sample, however, may reflect genetic stratification as well as random fluctuation of allele frequencies in the cases and controls, demonstrating the importance of large samples in which the phenotype is well assessed.

## 1. Introduction

Genetic influences play a major role in the development of alcohol use disorders as formal genetic studies in twins and epidemiological samples have shown [1,2,3]. Candidate studies and genome-wide association studies (GWAS) have identified numerous candidate genes for alcohol dependence (AD) and alcohol consumption (AC).

In candidate gene studies, the most consistently reported associations for both traits have been obtained for the genes alcohol dehydrogenase 1B (*ADH1B;* chr4q23) [4], alcohol dehydrogenase 1C (*ADH1C*; chr4q23) [5], and aldehyde dehydrogenase 2 (*ALDH2*; chr12q24) [6]. 

The advantage of GWAS studies which screen the entire genome with millions of variants is that they facilitate gene identification in novel biological contexts. Indeed, recent successes have been achieved for complex traits with low heritability, such as depression [7] in large samples comprising more than one hundred thousand individuals. Previous GWAS of AD have been conducted in samples with a lower number of individuals [8,9,10]. However, they have also most consistently identified variants in the *ADH* gene cluster. The up-to-now largest GWAS of AC which contained more than one hundred thousand individuals from the UK Biobank, and identified ten loci reaching genome-wide significance, also showed the best finding in the *ADH1B/ADH1C* region (rs145452708, *p* = 8.93 × 10^−29^) [11]. Additionally, earlier GWAS of AC identified rs1229984 and other variants at the chr4q22/q23 region in/near the *ADH* gene cluster [12,13,14].

A promising approach to mitigating the burden of multiple testing, which limits the single-marker approach, is to analyze the aggregated contribution of variants in single genes and in functionally related gene groups (e.g., biological pathways), under the assumption that these contain a large number of variants with a disruptive influence on gene/pathway function.

In the present study, multi-marker analyses were performed in order to detect new genes and pathways for AD and/or to confirm prior reported genes and pathways. To increase sample size in order to maximize statistical power, individuals with alcohol-related somatic disease were also included. Gene-wide significance for the *ADH1B* gene was detected in the combined sample. However, the findings demonstrated that very large sample sizes are warranted to overcome heterogeneity and/or random genetic fluctuation. 

## 2. Materials and Methods

### 2.1. Participants

The cohort comprised individuals from three previously reported German samples: (i) 1331 AD patients and 1934 controls [9]; (ii) 1110 patients with chronic alcoholic pancreatitis (ACP) [15]; and (iii) 400 patients with alcohol-related liver cirrhosis (ALC) [16]. A control sample (*n* = 1750) for the ACP + ALC cases was drawn from the Heinz Nixdorf Recall (HNR) study [17].

The study was approved by the ethics committee II of Medical Faculty Mannheim of Heidelberg University study number 2012-361N-MA, and was carried out in accordance with the Declaration of Helsinki. All subjects provided written informed consent prior to inclusion.

Alcohol dependence (AD) case-control subsample: A detailed description of the sample is provided elsewhere [9]. All patients were of self-reported German ancestry, and fulfilled Diagnostic and Statistical Manual of Mental Disorders, 4th Edition (DSM-IV) criteria for AD [18]. The patients were recruited from consecutive admissions to psychiatric units at university hospitals participating in the German Addiction Research Network [19]. These five study centers are located in the following areas of central and southern Germany: Mannheim, Bonn/Essen/Düsseldorf/Homburg, Regensburg, Munich, and Mainz. Controls were drawn from the following three population-based epidemiological cohorts: (i) KORA-gen [20]; (ii) PopGen [21]; and (iii) HNR [17]. Further controls were drawn at random from a Munich community sample screened using the Composite International Diagnostic Interview. The AD case-control subsample is part of the Psychiatric Genomics Consortium (PGC) [22]. The GWAS data from the samples can be made available within the context of research collaborations.

Chronic alcoholic pancreatitis (ACP) patients: A diagnosis of ACP was assigned in patients with a history of ≥2 years ingestion of ≥80 g alcohol/day (men), or ≥60 g/day (women). Most patients exceeded these cut-offs for level and/or duration. The cohort included patients from a number of European countries [15]. However, only German ACP patients were included in the present analyses. These individuals were recruited in Berlin, Dresden, Erlangen, Heidelberg, Greifswald, Leipzig, Magdeburg, Mannheim, and Munich.

Alcohol-related liver cirrhosis (ALC) patients: A detailed description of the criteria used to define case status is provided in [16]. ALC patients presented with clinically-diagnosed, or biopsy-confirmed, cirrhosis and a ≥10-year history of a past and/or present alcohol consumption level of ≥80 g/day (men), or ≥60 g/day (women). In all cases, other causes of cirrhosis were excluded. ALC cases were recruited from university hospital departments of hepatology and gastroenterology in: (i) Germany (Bonn, Regensburg, Dresden, Leipzig, Kiel, Frankfurt); (ii) Austria (Salzburg); and (iii) Switzerland (Bern).

Controls for ACP and ALC patients: Controls were drawn from the HNR study [17].

### 2.2. Genotyping

AD case-control subsample: Patient samples were individually genotyped using the Illumina HumanHap550, Human610Quad, or Human660w Quad BeadChips (Illumina, Inc., San Diego, CA, USA). Controls were genotyped using the Illumina HumanHap550 Bead Chip.

ACP patient subsample, ALC patient subsample, and control sample for ACP and ALC patients: The individuals were genotyped using the Illumina Omni Express BeadChip.

### 2.3. Quality Control

Quality control (QC) and single marker association testing were performed using PLINK v1.9 [23]. Prior to QC, the genotype data of the three samples were merged. QC of the merged data was performed in accordance with the protocol of the Schizophrenia Working Group of the Psychiatric Genomics Consortium [24]. The analyses were restricted to autosomal single nucleotide polymorphisms (SNPs) only. In the combined sample, genetic outliers were identified using principal component analysis (PCA). Outlier status was defined as the presence of data points located more than 6 standard deviations from the mean on any of the first 20 principal components. The respective individuals were excluded from further analysis. After the removal of PCA outliers, the first and second principal components showed a nominally significant association with AD, and were included as covariates in all association analyses. As the present sample was comparatively small, a stringent Hardy–Weinberg Equilibrium (HWE) test cutoff of *p* > 0.05 was applied to the controls of the combined sample in order to optimize the quality of the genotyping clusters for the purposes of multimarker analysis. A total of 6525 (out of 6894) individuals and 257,866 variants passed all filters. Of the 6525 individuals, 2841 were cases (1331 AD patients, 1110 ACP patients, 400 ALC patients), and 3684 were controls (1934 controls from the AD subsample, and 1750 controls for the combined ACP and ALC patient sample). After linkage disequilibrium (LD)-based pruning (Variance Inflation Factor of 10), a total of 194,024 SNPs remained for the combined sample and subsample analyses.

### 2.4. Statistical Analysis

The single marker analysis was conducted using PLINK v1.9 [23]. This involved logistic regression, an additive model of inheritance and correction for population stratification by including the first two principal components as covariates. Gene-based and pathway-based analysis was conducted using MAGMA v1.04 [25]. For the pathway analysis, output files from the gene-based analysis were used as input. SNPs were assigned to a gene if the variant was located within the gene sequence or within 20 kb of the transcript. If a variant was located within a region shared by more than one gene, the variant was assigned to all of the respective genes. Version 5.1 of the Reactome database set was retrieved from the Molecular Signatures Database [26]. The Reactome v5.1 set comprises 674 pathways.

For the post hoc analysis, genotypes for the *ADH* variant rs1789891 were counted using the “hardy” option in PLINK. Deviation from HWE (exact test) was calculated using the DeFinetti program [27]. SNAP [28] was used to generate the regional association plot. The database dbSNP [29] was used to retrieve the nucleotide triplet for the amino acid exchange.

## 3. Results

A single marker analysis in the combined sample generated no genome-wide significant findings (Table S1). The top five variants were: (i) rs10392 (*p* = 8.336 × 10^−7^; Odds Ratio (OR) = 1.252), located in *PPP1R16B* (protein phosphatase 1 regulatory subunit 16B), and near *FAM83D* (family with sequence similarity 83 member D); (ii) rs454510 (3.334 × 10^−6^; OR = 1.206) near zinc finger protein 697 (*ZNF697*); (iii) rs2028201 (4.928 × 10^−6^; OR = 1.183) in spermatogenesis associated serine rich 2 like (*SPATS2L*); (iv) intergenic variant rs926544 (*p* = 1.133 × 10^−^^5^; OR = 0.8113) and (v) rs1789891 (*p* = 1.315 × 10^−^^5^; OR = 1.232) near alcohol dehydrogenase 1B (*ADH1B*) and near alcohol dehydrogenase 1C (*ADH1C*).

In the gene-based approach, only one finding achieved genome-wide significance. This was the association with alcohol dehydrogenase 1B (*ADH1B*) (*p*_uncorrected_ = 1.2 × 10^−6^; *p*_corrected_ = 0.020; Table 1). The Bonferroni corrected gene-based genome-wide significance threshold was 2.9 × 10^−6^ (0.05/16853 genes). In the combined genotype data, 16,853 genes were represented. Table 2 shows the variants of the *ADH1B* locus that were included in the gene-based analysis. This association was driven by the AD subsample (AD: *p*_uncorrected_ = 6.5 × 10^−10^; ACP + ALC: *p*_uncorrected_ = 0.69).

The *ADH* variant which made the strongest contribution to the gene-wide significance of *ADH1B* was rs1789891, which is located within ±20 kb of *ADH1B* and *ADH1C* and contributes to the gene-based *p*-values of both genes (Figure S1). The rs1789891 association was driven by the AD subsample (combined sample: *p* = 1.315 × 10^−5^, OR_A-allele_ = 1.232; ACP + ALC subsample: *p* = 0.6392, OR_A-allele_ = 1.033; AD subsample: *p* = 1.642 × 10^−8^, OR_A-allele_ = 1.469) (Table S1). The rs1789891 “risk” allele was A and the “protective” allele was C.

In the control population of the combined cohort, the rs1789891 allele frequency was 0.154 for the A-allele. The A-allele frequency of the controls was 0.141 for the AD subsample and 0.169 for the ACP + ALC subsample (Table 3), the latter being similar to that reported in the 1000 Genomes Phase 3 data (A-allele: 0.167; CEU subpopulation) [30].

In the pathway analysis of the combined sample, the top finding was the “Ethanol_Oxidation” gene set, which contains *ADH1B* (*p*_uncorrected_ = 2.2 × 10^−4^; *p*_corrected_ = 0.15; Table 4). 

## 4. Discussion

To facilitate both the identification of new genes and pathways for AD and the replication of previous results, the present study combined the cohorts of three previous investigations in order to increase sample size to increase statistical power. The only association to withstand correction for multiple testing was the association with *ADH1B* in the gene-based test. This association was attributable to the AD subsample, and no association was detected in the ACP + ALC subsample. On first inspection, the lack of association with *ADH1B* in the combined ACP + ALC sample may seem surprising, since this gene is one of most consistently reported genes for AD and AC, and achieved genome-wide significance in the present AD subsample. In the AD patient subsample, the frequency of the A allele was higher (19.4% vs. 17.9%), and in the controls lower (14.1% vs. 16.9%) than in the ACP + ALC subsample. The lack of association may be attributable to random or systematic genetic differences within the patient and/or control samples. 

### 4.1. Patients

1. For ACP and ALC, no explicit diagnosis of AD was required, and these patients may therefore differ in terms of genetic disposition. However, the ACP and ALC patients were recruited from a clinic specialized in the treatment of alcohol-induced somatic disorders. Furthermore, in each patient, the respective disorder had been induced by excessive alcohol consumption, and the majority of patients were unable to abstain from alcohol despite the assignment of the somatic diagnosis.
2. The differing distribution of rs1789891 in the AD and ACP + ALC samples is non-random. *ADH1B* metabolizes alcohol to acetaldehyde, and research suggests that the adverse effects of acetaldehyde inhibit further drinking [31,32,33]. Alleles that confer an increased rate of alcohol metabolism may also contribute to tissue damage [34]. This was illustrated in a recent study from Japan, which analyzed rs1229984 (Arg48His) in *ADH1B*. The *ADH1B* 48His variant leads to an increased level of acetaldehyde and is thus protective in terms of AD development. The authors found that the *ADH1B* 48His variant was overrepresented in patients with alcoholic liver cirrhosis and chronic alcoholic calcific pancreatitis [35]. *ADH1B*_48His has a low frequency in Europeans [36,37,38], was not present in our genotyping arrays, and could not be imputed with sufficient imputation quality (R^2^ = 0.44).

In the present analyses, the *ADH1B* variant with the lowest *p*-value was rs1789891 (*p* = 1.315 × 10^−5^). The rs1789891 variant is located between the genes *ADH1B* and *ADH1C*. Although both genes are expressed in the liver [39] and pancreas [40], rs1789891 has no known function according to the NCBI Phenotype-Genotype Integrator [41]. However, rs1789891 is in high LD with the functional variants rs1693482 (Arg272Gln) and rs698 (Ile350Val) in the gene *ADH1C* (Figure S2). These two *ADH1C* missense variants Arg272Gln and Ile350Val typically occur together (r^2^ = 1.0), and result in two different forms of ADH1C: (i) the ADH1C isoenzyme gamma1 (ADH1C*1), in which arginine is present at amino acid position 272 and isoleucine at amino acid position 350; and (ii) the ADH1C isoenzyme gamma2 (ADH1C*2), in which glutamine is present at amino acid position 272 and valine at amino acid position 350. Although *ADH1C*_Arg272/Ile350 (which correspond to the rs1789891 C-allele) confers a rapid rate of ethanol oxidation [42,43], its effect on the rate of alcohol metabolism is weaker than that of *ADH1B*_48His. However, imputation of rs1693482 and rs698 showed that the associations with rs1693482 (*p* = 1.43 × 10^−5^) and rs698 (*p* = 1.798 × 10^−5^) were weaker than with rs1789891 (*p* = 1.315 × 10^−5^). Thus, the issue of whether the association with rs1789891 is mainly attributable to LD with rs1693482 and rs698, or whether further variants in this region are implicated, remains unclear. Variants rs1693482/rs698 could nevertheless be the contributory factor in terms of organ damage [44,45,46], since they are in LD with rs1789891. 

Two plausible hypotheses can be formulated to explain how the products of the ADH reaction may increase the risk of tissue damage in the pancreas and liver. First, acetaldehyde accumulation in response to chronic alcohol ingestion has been implicated in the etiology of liver cirrhosis, pancreatitis, brain damage, cardiomyopathy, fetal alcohol syndrome, and various forms of cancer [34]. Whereas the allele or genotype differences in some studies were non-significant, several investigations have reported a lower frequency of alcoholism-susceptibility alleles or genotypes in patients with alcoholic liver disease or alcoholic pancreatitis (reviewed in [35]). The cytotoxic acetaldehyde that is formed as an intermediate in the metabolism of ethanol is reported to induce morphological changes in the pancreas of experimental animals [47]. In addition, acetaldehyde has reported fibrogenic effects in the liver [48]. The likely molecular mechanism through which acetaldehyde causes organ damage is the promotion of adduct formation, which leads to protein and DNA damage [48].

Second, nicotinamide adenine dinucleotide (NAD^+^) is an intermediate electron carrier in the cytosolic ADH-mediated metabolism of alcohol to acetaldehyde. In this reaction, NAD^+^ is reduced to NADH by two electrons. In a subsequent step, the electrons of NADH are transferred to O_2_ in the mitochondrial respiratory chain, which captures H^+^ to yield H_2_O. Ethanol metabolism therefore increases the O_2_ requirement of hepatocytes, and may result in hepatocyte hypoxia [49]. This may lead in turn to organ damage.

### 4.2. Controls

Random fluctuation seems very likely when looking at allele frequencies in the control samples. A detailed inspection of rs1789891 allele frequencies in the present control subsamples (data not shown) was therefore performed. Despite the fact that the control sample used for the ALC + ACP samples was drawn from the same study as a subcohort of the AD controls, it displayed a higher frequency of the AD risk allele A (16.9%) than the corresponding control subcohort used for AD (13.9%).

## 5. Conclusions

The aim of the present study was to identify new genes and pathways for AD, and to confirm previously reported findings, by combining three previously investigated samples. No novel data were generated. The fact that the previously reported association with *ADH1B* was not observed in the ACP + ALC subsample may reflect genetic stratification in cases and/or random fluctuation in allele frequencies in controls. This finding demonstrates that even strong signals can be blurred, if samples are small and heterogeneous with possible opposing effects. Our finding therefore stresses the necessity for, and central importance of samples that are large and well characterized, such as those investigated within the context of the Psychiatric Genomics Consortium. The present authors are optimistic that as has been the case with other psychiatric disorders, the possibilities offered by GWAS will ultimately generate major contributions to our understanding of the genetic background of the alcohol use disorders.

## Figures and Tables

**Table 1:** Results of the gene-based analysis in the combined sample, and the respective *p*-values in the subsamples. Genes with *p*_uncorrected_ < 1.0 × 10^−4^ are shown. If a variant was located within a region shared by more than one gene, the variant was assigned to all of the respective genes.

Gene	Position (hg18)	*p*-Value in the Combined Sample (2841 Cases, 3684 Controls)	*p*-Value in the ACP + ALC Subsample (1510 Cases, 1750 Controls)	*p*-Value in the AD Subsample (1331 Cases, 1934 Controls)
Alcohol Dehydrogenase 1B (*ADH1B*)	chr4: 100426552-100481581	1.209 × 10^−6^	0.68776	6.5258 × 10^−10^
Family with Sequence Similarity 83 Member D (*FAM83D*)	chr20: 36968369-37035117	4.2443 × 10^−6^	0.0070016	0.34143
Alcohol Dehydrogenase 1C (*ADH1C*)	chr4: 100456672-100512940	9.9601 × 10^−6^	0.86648	8.5406 × 10^−10^
Zinc finger protein 697 (*ZNF697*)	chr1: 119943523-120011913	2.3488 × 10^−5^	0.0011435	0.0087101
Ras Homolog Family Member T2 (*RHOT2*)	chr16: 638134-684172	3.006 × 10^−5^	0.025274	0.00029276
Crystallin Zeta (*CRYZ*)	chr1: 74923772-74991315	3.0976 × 10^−5^	0.051974	0.00084007
X-ray repair cross complementing 5 (*XRCC5*)	chr2: 216662378-216799248	3.4649 × 10^−5^	0.27561	2.7093 × 10^−5^
COX14 Cytochrome C Oxidase Assembly Factor (*COX14*)	chr12: 48772167-48820501	3.7703 × 10^−5^	0.0017051	0.0044189
Rhomboid, Veinlet-Like 1 (*RHBDL1*)	chr16: 646076-688268	4.5926 × 10^−5^	0.026591	0.00033002
LOC440330 (*LOC440330*)	chr16: 648519-689772	4.5926 × 10^−5^	0.026591	0.00033002
STIP1 homology and U-box containing protein 1 (*STUB1*)	chr16: 650116-692769	4.5926 × 10^−5^	0.026591	0.00033002
Jumonji domain containing 8 (*JMJD8*)	chr16: 651668-694440	4.62 × 10^−5^	0.026732	0.00035589
WD Repeat Domain 24 (*WDR24*)	chr16: 654703-700401	4.62 × 10^−5^	0.026732	0.00035589
F-Box and Leucine-Rich Repeat Protein 16 (*FBXL16*)	chr16: 662503-715809	4.62 × 10^−5^	0.026732	0.00035589
Olfactomedin Like 2A (*OLFML2A*)	chr9: 126559258-126636982	4.8774 × 10^−5^	0.00044653	0.015794

AD: Alcohol dependence; ACP: Chronic alcoholic pancreatitis; ALC: Alcohol-related liver cirrhosis.

**Table 2:** Variants used as input for the gene-based analysis of *ADH1B* (±20 kb; chr4:100426552-100481581; hg18). Single marker association *p*_uncorrected_-values shown, as calculated using logistic regression in PLINK.

SNP (Genotypes)	*p*-Value in the Combined Sample (2841 Cases, 3684 Controls); [OR]; Genotype Counts : All (MAF), Affected (MAF), Unaffected (MAF)	*p*-Value in the ACP + ALC Subsample (1510 Cases, 1750 Controls); [OR]; Genotype Counts: All (MAF), Affected (MAF), Unaffected (MAF)	*p*-Value in the AD Subsample (1331 Cases, 1934 Controls); [OR]; Genotype Counts: All (MAF), Affected (MAF), Unaffected (MAF)
rs1159918 (TT/TG/GG)	0.6477	0.6256	0.271
	[1.018]	[0.9732]	[1.061]
	731/2941/2846 (0.3378)	360/1486/1407 (0.3391)	371/1455/1439 (0.3364)
	324/1285/1227 (0.3408)	169/679/657 (0.3379)	155/606/570 (0.3441)
	407/1656/1619 (0.3354)	191/807/750 (0.3401)	216/849/869 (0.3312)
rs1229982 (TT/TG/GG)	0.2306	0.6865	0.1655
	[0.947]	[0.9738]	[0.9153]
	262/2034/4229 (0.196)	127/1015/2118 (0.1946)	135/1019/2111 (0.1974)
	104/884/1853 (0.1922)	52/485/973 (0.195)	52/399/880 (0.189)
	158/1150/2376 (0.199)	75/530/1145 (0.1943)	83/620/1231 (0.2032)
rs9307239 (TT/TC/CC)	0.5449	0.5695	0.6132
	[0.9781]	[0.9707]	[0.974]
	1084/3105/2334 (0.4042)	581/1556/1121 (0.4171)	503/1549/1213 (0.3913)
	485/1307/1047 (0.401)	278/688/542 (0.4125)	207/619/505 (0.3881)
	599/1798/1287 (0.4066)	303/868/579 (0.4211)	296/930/708 (0.3935)
rs1789891 (AA/AC/CC)	1.315 × 10^−5^	0.6392	1.642 × 10^−8^
	[1.232]	[1.033]	[1.469]
	200/1782/4507 (0.1681)	106/910/2209 (0.174)	94/872/2298 (0.1624)
	102/850/1879 (0.1862)	49/440/1011 (0.1793)	53/410/868 (0.1938)
	98/932/2628 (0.1542)	57/470/1198 (0.1693)	41/462/1430 (0.1407)
rs2173201 (AA/AC/CC)	0.0005063	0.2231	0.0001093
	[0.8584]	[0.9274]	[0.7823]
	316/2286/3923 (0.2236)	172/1164/1924 (0.2313)	144/1122/1999 (0.2159)
	127/933/1781 (0.2089)	81/512/917 (0.2232)	46/421/864 (0.1927)
	189/1353/2142 (0.2349)	91/652/1007 (0.2383)	98/701/1135 (0.2319)

SNP: Single nucleotide polymorphism; BP: Base pair; OR: Odds ratio. MAF: Minor allele frequency.

**Table 3:** Genotype counts, allele frequencies, and Hardy–Weinberg Equilibrium (HWE) *p*-values for rs1789891 in the combined sample, and the respective *p*-values in the subsamples. Genotype counts for rs1789891 are shown; number of individuals with the respective rs1789891 genotype is shown.

Sample	Subgroup	Genotype Counts AA/AC/CC	Frequency of rs1789891 Risk Allele for AD (A-allele); Frequency of rs1789891 Protective Allele for AD (C-Allele)	*p* HWE
Combined sample (2831 cases, 3658 controls)	all	200/1782/4507	0.168; 0.832	0.14
	affected	102/850/1879	0.186; 0.814	0.62
	unaffected	98/932/2628	0.154; 0.846	0.16
ACP + ALC subsample (1500 cases, 1725 controls)	all	106/910/2209	0.174; 0.826	0.30
	affected	49/440/1011	0.179; 0.821	0.86
	unaffected	57/470/1198	0.169; 0.831	0.20
ACP subsample (1101 cases)	affected	35/320/746	0.177; 0.823	0.92
ALC subsample (399 cases)	affected	14/120/265	0.185; 0.815	0.87
AD subsample (1331 cases, 1933 controls)	all	94/872/2298	0.162; 0.838	0.30
	affected	53/410/868	0.194; 0.806	0.60
	unaffected	41/462/1430	0.141; 0.859	0.57

**Table 4:** Results of the genome-wide pathway analysis of the combined sample, and the respective values in the subsamples. Pathways with *p*_uncorrected_ < 1 × 10^−2^ are shown. If a variant was located within a region shared by more than one gene, the variant was assigned to all of the respective genes.

Pathway	NGENES	BETA of the Combined Sample	BETA_STD of the Combined Sample	SE in the Combined Sample	*p*–Value in the Combined Sample (2841 Cases, 3684 Controls)	*p*-Value in the ACP + ALC Subsample (1510 Cases, 1750 Controls)	*p*-Value in the AD Subsample (1331 Cases, 1934 Controls)
Ethanol Oxidation	10	1.28	0.0312	0.364	0.00021694	0.061561	9.15 × 10^−5^
Organic Cation Anion Zwitterion Transport	13	0.844	0.0234	0.267	0.00077746	0.025914	0.37297
Amino Acid Transport across the Plasma Membrane	28	0.429	0.0175	0.161	0.0037699	0.41315	0.52029
Basigin Interactions	23	0.428	0.0158	0.161	0.0038618	0.50705	0.17262
Recruitment of NUMA to Mitotic Centrosomes	9	0.746	0.0172	0.285	0.0044978	0.073773	0.27771
Trafficking and Processing of Endosomal TLR	9	0.739	0.0171	0.3	0.0068239	0.35393	0.313

BETA: Regression coefficient; STD: Standard deviation; SE: Standard error.

## Supplementary Materials

The following are available online at www.mdpi.com/2073-4425/8/7/183/s1. Figure S1: Regional association plot of the alcohol dehydrogenase gene region in the combined sample; Figure S2: Linkage disequilibrium between rs1789891 and rs1229984, rs1693482, and rs698. Table S1: Top SNPs from the single marker analysis in the combined sample and their respective *p*-values in the subsamples.
