# Supplementary material for: Genetic Contribution to Alcohol Dependence: Investigation of a Heterogeneous German Sample of Individuals with Alcohol Dependence, Chronic Alcoholic Pancreatitis, and Alcohol-Related Cirrhosis
Source: Genes (Basel). 2017 Jul 17;8(7):183. doi: 10.3390/genes8070183 (PMC5541316; doi:10.3390/genes8070183)
Supplement: Supplementary file 1 [file genes-08-00183-s001.pdf]

Supplementary Materials: Genetic Contribution to Alcohol Dependence: Investigation of a Heterogeneous German Sample of Individuals with Alcohol Dependence, Chronic Alcoholic Pancreatitis, and Alcohol-related Cirrhosis

Jens Treutlein, Josef Frank, Fabian Streit, Céline S. Reinbold, Dilafruz Juraeva, Franziska Degenhardt, Liz Rietschel, Stephanie H. Witt, Andreas J. Forstner, Monika Ridinger, Jana Strohmaier, Norbert Wodarz, Helene Dukal, Jerome C. Foo, Per Hoffmann, Stefan Herms, Stefanie Heilmann-Heimbach, Michael Soyka, Wolfgang Maier, Wolfgang Gaebel, Norbert Dahmen, Norbert Scherbaum, Bertram Müller-Myhsok, Susanne Lucae, Marcus Ising, Felix Stickel, Thomas Berg, Susanne Möbus, Karl-Heinz Jöckel, Henrike Scholz, Ulrich S. Zimmermann, Stephan Buch, Wolfgang H. Sommer, Rainer Spanagel, Benedikt Brors, Sven Cichon, Karl Mann, Falk Kiefer, Jochen Hampe, Jonas Rosendahl, Markus M. Nöthen, and Marcella Rietschel

Figure S1. Regional association plot of the alcohol dehydrogenase gene region in the combined sample. The strongest variant in this region, rs1789891, is located within +-20kb of *ADH1B* and *ADH1C*, and therefore contributes to the gene-based *P*-values of both genes.

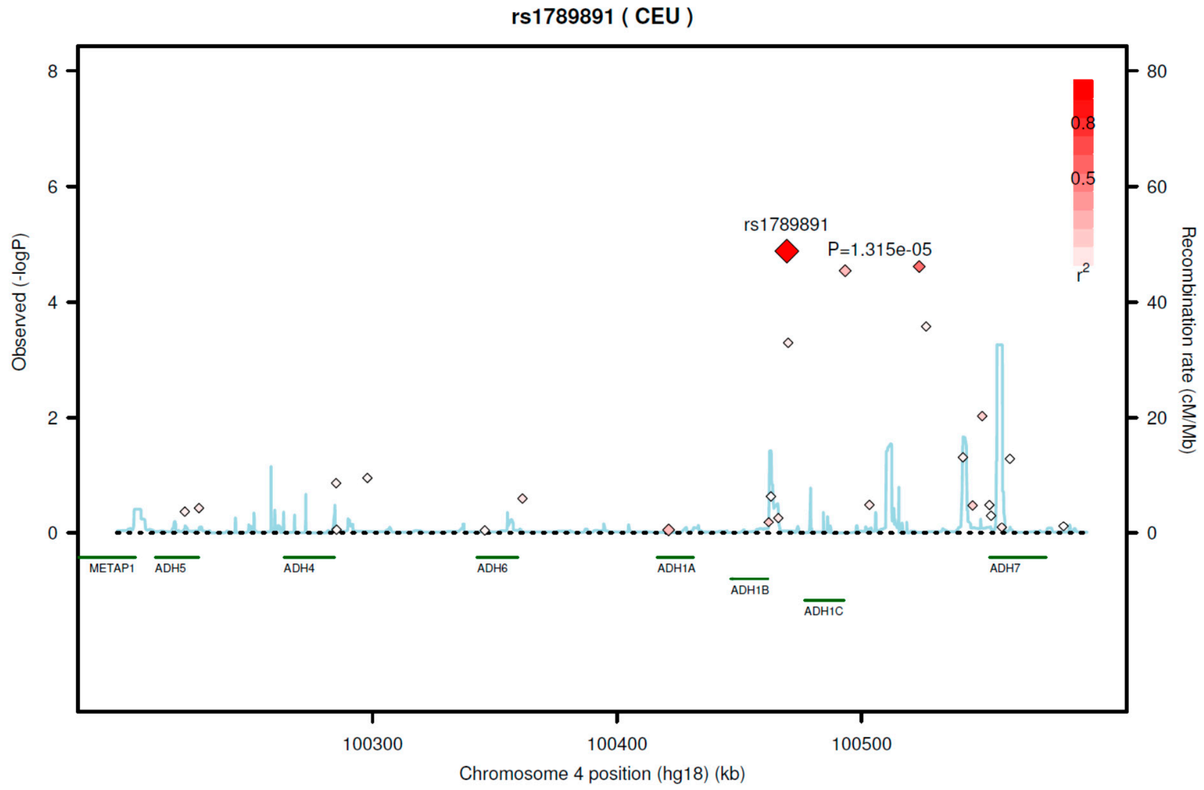

Figure S2. Linkage disequilibrium between: (a) rs1789891 and rs1229984 (*ADH1B*_Arg48His); (b) rs1789891 and rs1693482 (*ADH1C*_Arg272Gln); and (c) rs1789891 and rs698 (*ADH1C*_Ile350Val) (https://analysistools.nci.nih.gov/LDlink/?tab=ldpair; 1000 Genomes Phase 3 data).

(a) The rs1229984 A-allele containing nucleotide triplet codes for the amino acid histidine (His), resulting in an ADH1B enzyme with an increased rate of turnover (opposite strand sequence of the rs1229984 T-allele in the figure below: CAC coding for His; https://www.ncbi.nlm.nih.gov/projects/SNP/snp_ref.cgi?rs=1229984). Linkage disequilibrium between rs1789891 and rs1229984 is low.

rs1789891 | rs1229984 | CEU

		rs1229984 chr4:100239319 C	rs1229984 chr4:100239319 T	
rs1789891 chr4:100250419	A	32	1	33 (0.167)
	C	163	2	165 (0.833)
		195 (0.985)	3 (0.015)	198

Haplotypes	
C_C:	163 (0.823)
A_C:	32 (0.162)
C_T:	2 (0.01)
A_T:	1 (0.005)

Statistics	
D':	0.2
R^2:	0.0031
Chi-sq:	0.6092
p-value:	0.4351

rs1789891 and rs1229984 are in linkage equilibrium

(b) The rs1693482 C-allele containing nucleotide triplet codes for the amino acid arginine (Arg), which results in an ADH1C enzyme with an increased rate of turnover (opposite strand sequence of the rs1693482 C-allele in the figure below: C**G**G coding for Arg; https://www.ncbi.nlm.nih.gov/projects/SNP/snp_ref.cgi?rs=1693482). The rs1789891 "protective" C-allele is correlated with the rs1693482 "protective" C-allele.

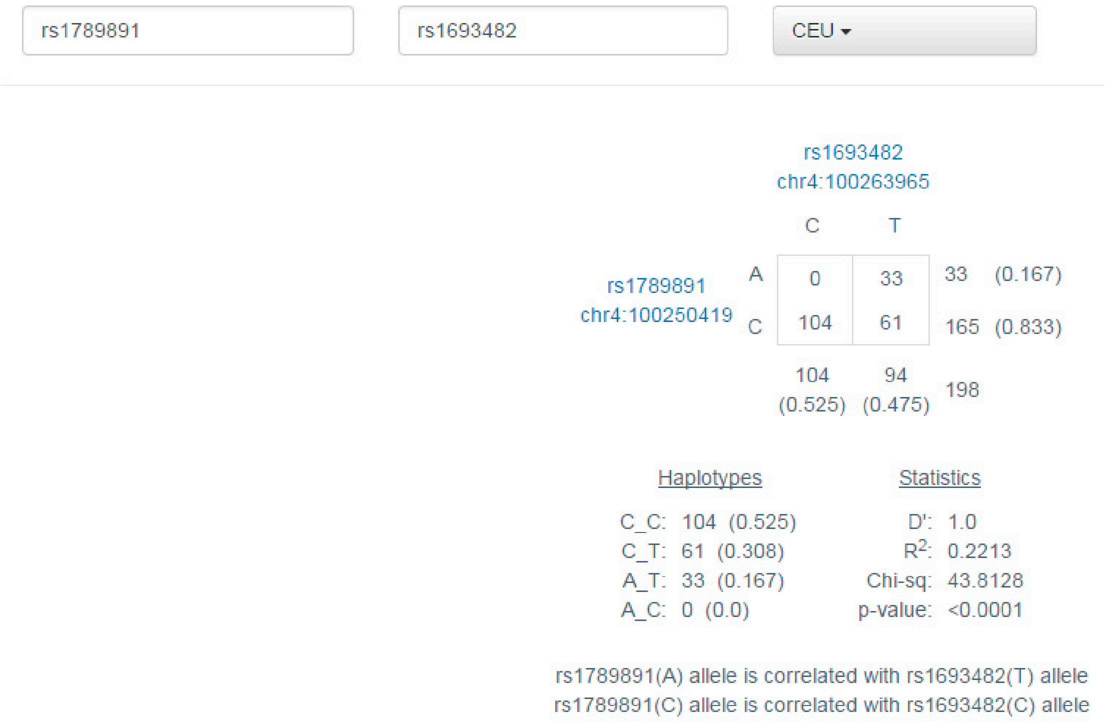

(c) The rs698 A-allele containing nucleotide triplet codes for the amino acid isoleucine (Ile), which results in an ADH1C enzyme with an increased rate of turnover (opposite strand sequence of the rs698 T-allele in the figure below: **A**TT coding for Ile; https://www.ncbi.nlm.nih.gov/projects/SNP/snp_ref.cgi?rs=698). The rs1789891 "protective" C-allele is correlated with the rs698 "protective" T-allele.

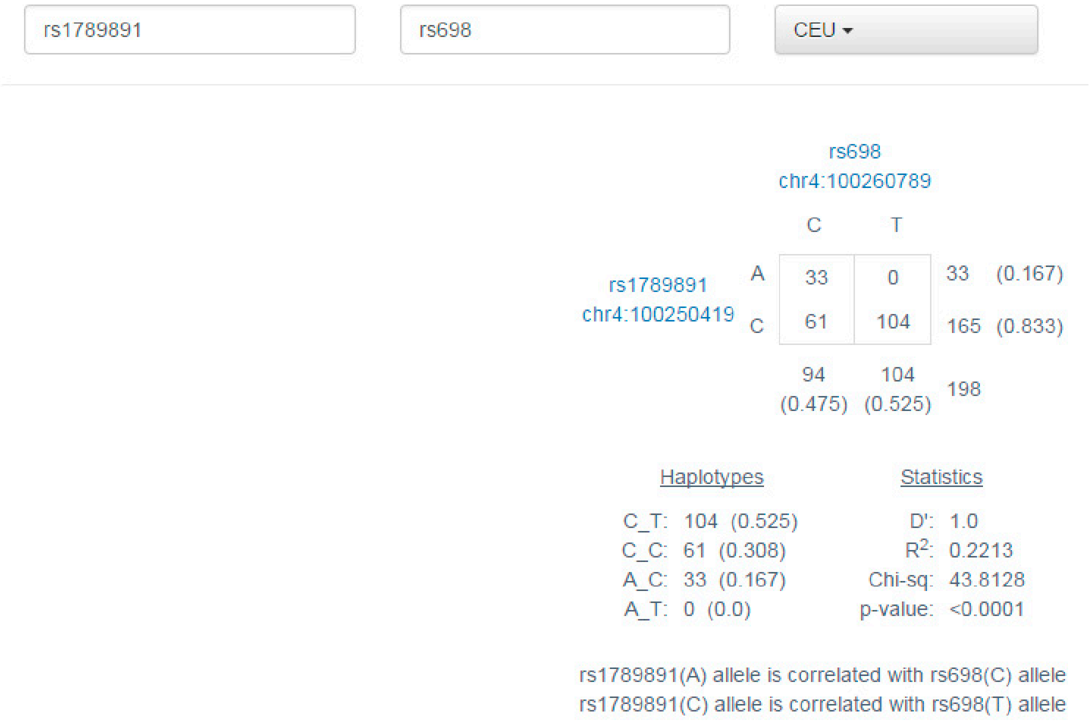

Table S1. Top SNPs from the single marker analysis in the combined sample, and their respective P-values in the subsamples. SNPs with $P_{uncorrected}$ <1.00e-04 are shown. SNP: Single Nucleotide Polymorphism; OR: Odds Ratio. MAF: minor allele frequency; AD: alcohol dependence; ACP: chronic alcoholic pancreatitis; ALC: alcohol-related cirrhosis; Chr: chromosome.

**Gene (RefSeq)**	**Chr**	**SNP**	**Position**	**Effect allele in the combined sample**	**Combined sample** (2841 cases, 3684 controls);		**ACP+ALC subsample** (1510 cases, 1750 controls)		**AD subsample** (1331 cases, 1934 controls)	
					P-Value [OR]	Genotype counts: All (MAF), Affected (MAF), Unaffected (MAF)	***P-Value [OR]***	Genotype counts: All (MAF), Affected (MAF), Unaffected (MAF)	***P-Value [OR]***	Genotype counts: All (MAF), Affected (MAF), Unaffected (MAF)
Protein phosphatase 1 regulatory subunit 16B (*PPP1R16B*) Family With Sequence Similarity 83 Member D (*FAM83D*, near)	20	rs10392	36984349	A	8.336e-07 [1.252]	242/2049/4232 (0.1942) 140/927/1774 (0.2124) 102/1122/2458 (0.1801)	0.002328 [1.219]	132/1031/2095 (0.1987) 75/498/937 (0.2146) 57/533/1158 (0.1851)	0.0002549 [1.268]	110/1018/2137 (0.1896) 65/429/837 (0.21) 45/589/1300 (0.1755)
Zinc finger protein 697 (*ZNF697*, near)	1	rs454510	119996565	A	3.334e-06 [1.206]	512/2699/3309 (0.2855) 241/1251/1346 (0.3053) 271/1448/1963 (0.2702)	1.175e-05 [1.294]	245/1358/1653 (0.2838) 129/678/701 (0.3103) 116/680/952 (0.2609)	0.05006 [1.117]	267/1341/1656 (0.2872) 112/573/645 (0.2996) 155/768/1011 (0.2787)
Spermatogenesis associated serine rich 2 like (*SPATS2L*)	2	rs2028201	201011255	T	4.928e-06 [1.183]	1041/3063/2411 (0.3949) 508/1363/969 (0.4188) 533/1700/1442 (0.3763)	0.001659 [1.182]	518/1541/1198 (0.3956) 263/740/506 (0.4195) 255/801/692 (0.375)	0.0008096 [1.189]	523/1522/1213 (0.3941) 245/623/463 (0.4181) 278/899/750 (0.3775)
Intergenic	22	rs926544	37901380	T	1.133e-05 [0.8113]	221/1865/4431 (0.177) 87/742/2010 (0.1613) 134/1123/2421 (0.1891)	0.04158 [0.8704]	112/906/2237 (0.1736) 46/403/1060 (0.164) 66/503/1177 (0.1818)	4.003e-05 [0.7576]	109/959/2194 (0.1804) 41/339/950 (0.1583) 68/620/1244 (0.1957)
Alcohol Dehydrogenase 1B (*ADH1B*, near), Alcohol Dehydrogenase 1C (*ADH1C*, near)	4	rs1789891	100469442	A	1.315e-05 [1.232]	200/1782/4507 (0.1681) 102/850/1879 (0.1862) 98/932/2628 (0.1542)	0.6392 [1.033]	106/910/2209 (0.174) 49/440/1011 (0.1793) 57/470/1198 (0.1693)	1.642e-08 [1.469]	94/872/2298 (0.1624) 53/410/868 (0.1938) 41/462/1430 (0.1407)
Long non-coding RNA STX18 antisense RNA 1 (*STX18-AS1*, near)	4	rs1000579	4770395	G	1.471e-05 [1.172]	1176/3195/2151 (0.4253) 569/1414/858 (0.4491) 607/1781/1293 (0.4068)	0.4327 [1.042]	600/1567/1090 (0.4248) 285/742/483 (0.4344) 315/825/607 (0.4164)	1.638e-07 [1.315]	576/1628/1061 (0.4257) 284/672/375 (0.4658) 292/956/686 (0.3981)
CD5 molecule like (*CD5L*)	1	rs2765501	156071272	A	1.539e-05 [0.8522]	1244/3284/1996 (0.4424) 494/1419/927 (0.4238) 750/1865/1069 (0.4567)	0.01413 [0.8782]	648/1646/965 (0.4514) 277/770/462 (0.4387) 371/876/503 (0.4623)	0.0003157 [0.828]	596/1638/1031 (0.4334) 217/649/465 (0.4068) 379/989/566 (0.4517)
Intergenic	12	rs1846035	82545653	G	1.542e-05 [0.8499]	885/2957/2663 (0.3633) 357/1243/1225 (0.3464) 528/1714/1438 (0.3764)	0.0011 [0.8389]	466/1508/1278 (0.3752) 204/678/622 (0.361) 262/830/656 (0.3873)	0.001861 [0.8472]	419/1449/1385 (0.3515) 153/565/603 (0.3297) 266/884/782 (0.3665)

SATB homeobox 2 (*SATB2)*	2	rs3828186	199922518	C	1.581e-05<br>[0.7736]	73/1304/5147 (0.1111)<br>27/483/2330 (0.09454)<br>46/821/2817 (0.1239)	0.0001147<br>[0.7135]	33/636/2590 (0.1077)<br>12/236/1261 (0.08615)<br>21/400/1329 (0.1263)	0.03371<br>[0.8408]	40/668/2557 (0.1145)<br>15/247/1069 (0.1041)<br>25/421/1488 (0.1218)
STIP1 homology and U-box containing protein 1 (*STUB1*); Jumonji domain containing 8 (*JMJD8)*	16	rs6597	671726	G	1.741e-05<br>[1.247]	143/1543/4828 (0.1404)<br>69/738/2030 (0.1544)<br>74/805/2798 (0.1296)	0.06735<br>[1.143]	73/814/2363 (0.1477)<br>36/396/1075 (0.1553)<br>37/418/1288 (0.1411)	4.605e-05<br>[1.347]	70/729/2465 (0.1331)<br>33/342/955 (0.1534)<br>37/387/1510 (0.1192)
Cdk5 and Abl enzyme substrate 1 (*CABLES1*, near)	18	rs11874608	18954411	T	2.132e-05<br>[0.8563]	1484/3280/1756 (0.4791)<br>593/1431/815 (0.4609)<br>891/1849/941 (0.4932)	0.0005777<br>[0.8355]	742/1613/901 (0.4756)<br>310/751/448 (0.4543)<br>432/862/453 (0.494)	0.01871<br>[0.8855]	742/1667/855 (0.4827)<br>283/680/367 (0.4684)<br>459/987/488 (0.4925)
Unc-51 like kinase 4 (*ULK4)*	3	rs6809441	41469609	C	2.42e-05<br>[0.8471]	641/2865/3019 (0.3178)<br>237/1222/1382 (0.2985)<br>404/1643/1637 (0.3327)	0.1328<br>[0.919]	324/1440/1496 (0.3202)<br>134/674/702 (0.3119)<br>190/766/794 (0.3274)	5.745e-06<br>[0.7757]	317/1425/1523 (0.3153)<br>103/548/680 (0.2832)<br>214/877/843 (0.3374)
Intergenic	4	rs1372680	100523568	A	2.425e-05<br>[1.284]	72/1191/5261 (0.1023)<br>33/589/2219 (0.1153)<br>39/602/3042 (0.09232)	0.2557<br>[1.101]	36/613/2610 (0.1051)<br>12/313/1185 (0.1116)<br>24/300/1425 (0.09949)	2.627e-06<br>[1.482]	36/578/2651 (0.09954)<br>21/276/1034 (0.1195)<br>15/302/1617 (0.08583)
Alcohol Dehydrogenase 1C (*ADH1C*, near)	4	rs1789924	100493309	T	2.869e-05<br>[1.166]	1127/3166/2231 (0.4154)<br>525/1427/888 (0.4361)<br>602/1739/1343 (0.3994)	0.5368<br>[1.033]	562/1600/1097 (0.4179)<br>266/746/497 (0.4235)<br>296/854/600 (0.4131)	1.274e-07<br>[1.314]	565/1566/1134 (0.4129)<br>259/681/391 (0.4504)<br>306/885/743 (0.387)
Long intergenic non-protein coding RNA 598 transcript variant TTL-B1 (*LINC00598*, near)	13	rs9549180	39811462	T	2.945e-05<br>[0.8277]	270/2195/4056 (0.2097)<br>83/921/1835 (0.1914)<br>187/1274/2221 (0.2238)	0.0001005<br>[0.7757]	134/1094/2028 (0.2092)<br>40/486/982 (0.1877)<br>94/608/1046 (0.2277)	0.04086<br>[0.878]	136/1101/2028 (0.2103)<br>43/435/853 (0.1957)<br>93/666/1175 (0.2203)
Ceramide synthase 5 (*CERS5)*	12	rs2242507	48813081	A	3.41e-05<br>[0.8408]	427/2502/3586 (0.2576)<br>150/1066/1620 (0.2408)<br>277/1436/1966 (0.2705)	0.001482<br>[0.8271]	222/1258/1779 (0.2611)<br>81/581/848 (0.246)<br>141/677/931 (0.2742)	0.004353<br>[0.844]	205/1244/1807 (0.254)<br>69/485/772 (0.2349)<br>136/759/1035 (0.2671)
X-ray repair cross complementing 5 (*XRCC5)*	2	rs828704	216701856	C	3.44e-05<br>[1.202]	277/2192/4049 (0.2106)<br>140/1020/1681 (0.2288)<br>137/1172/2368 (0.1966)	0.02829<br>[1.153]	129/1099/2032 (0.20819<br>77/514/919 (0.2212)<br>52/585/1113 (0.1969)	0.0001554<br>[1.263]	148/1093/2017 (0.2132)<br>63/506/762 (0.2374)<br>85/587/1255 (0.1964)
Zinc finger protein 697 (*ZNF697)*	1	rs838989	119984429	G	4.062e-05<br>[1.17]	752/2892/2871 (0.3374)<br>351/1313/1177 (0.3546)<br>401/1579/1694 (0.324)	0.0001748<br>[1.23]	369/1426/1465 (0.3319)<br>189/692/629 (0.3543)<br>180/734/836 (0.3126)	0.05974<br>[1.106]	383/1466/1406 (0.3429)<br>162/621/548 (0.355)<br>221/845/858 (0.3345)
Calpain 9 (*CAPN9)*	1	rs6677009	228973577	C	4.091e-05<br>[0.8456]	493/2697/3335 (0.2822)<br>194/1122/1525 (0.2658)<br>299/1575/1810 (0.2949)	0.004703<br>[0.8456]	228/1332/1700 (0.2742)<br>97/587/826 (0.2586)<br>131/745/874 (0.2877)	0.003944<br>[0.8483]	265/1365/1635 (0.2902)<br>97/535/699 (0.2739)<br>168/830/936 (0.3014)
Tumor necrosis factor superfamily member 18 (*TNFSF18)*	1	rs1883478	171251854	G	4.205e-05<br>[0.8591]	1011/3085/2427 (0.3915)<br>400/1317/1124 (0.3726)<br>611/1768/1303 (0.406)	0.001255<br>[0.8416]	495/1535/1230 (0.3873)<br>207/702/601 (0.3695)<br>288/833/629 (0.4026)	0.01027<br>[0.8748]	516/1550/1197 (0.3956)<br>193/615/523 (0.376)<br>323/935/674 (0.4092)

Fatty acid amide hydrolase (*FAAH*, near);<br>Fatty acid amide hydrolase pseudogene 1 (*FAAHP1*, near)	1	rs4660928	46658527	A	5.841e-05<br>[0.8494]	510/2664/3351 (0.2823)<br>195/1109/1537 (0.2638)<br>315/1555/1814 (0.2966)	0.000651<br>[0.82]	262/1356/1642 (0.2883)<br>102/606/802 (0.2682)<br>160/750/840 (0.3057)	0.01678<br>[0.8716]	248/1308/1709 (0.2763)<br>93/503/735 (0.2588)<br>155/805/974 (0.2883)
Neuroblastoma breakpoint family member 10 (*NBPF10*)	1	rs999095	145676851	G	6.194e-05<br>[1.229]	141/1539/4844 (0.1396)<br>68/745/2028 (0.1551)<br>73/794/2816 (0.1276)	0.000343<br>[1.316]	53/778/2428 (0.1356)<br>29/408/1073 (0.1543)<br>24/370/1355 (0.1195)	0.03316<br>[1.161]	88/761/2416 (0.1435)<br>39/337/955 (0.1559)<br>49/424/1461 (0.135)
Guanine deaminase (*GDA)*	9	rs17494172	74008904	G	6.956e-05<br>[0.7768]	58/1081/5385 (0.09174)<br>22/422/2397 (0.08201)<br>36/659/2988 (0.09924)	0.01125<br>[0.7906]	27/513/2720 (0.08696)<br>11/219/1280 (0.0798)<br>16/294/1440 (0.09314)	0.003083<br>[0.7703]	31/568/2665 (0.09651)<br>11/203/1117 (0.08452)<br>20/365/1548 (0.1048)
SATB homeobox 2 (*SATB2)*	2	rs6752494	200021568	C	7.122e-05<br>[0.8077]	131/1490/4880 (0.1347)<br>51/565/2215 (0.1178)<br>80/925/2665 (0.1478)	0.001301<br>[0.7777]	66/717/2453 (0.1312)<br>27/276/1197 (0.11)<br>39/441/1256 (0.1495)	0.02233<br>[0.8431]	65/773/2427 (0.1383)<br>24/289/1018 (0.1266)<br>41/484/1409 (0.1463)
Collagen type XXII alpha 1 chain (*COL22A1)*	8	rs11166844	139848062	C	7.172e-05<br>[0.8635]	1070/3139/2303 (0.4053)<br>428/1327/1078 (0.3853)<br>642/1812/1225 (0.4208)	0.002661<br>[0.8516]	520/1578/1152 (0.4028)<br>221/712/572 (0.3834)<br>299/866/580 (0.4195)	0.008264<br>[0.8721]	550/1561/1151 (0.4079)<br>207/615/506 (0.3874)<br>343/946/645 (0.4219)
Membrane spanning 4-domains A4A (*MS4A4A*, near);<br>Membrane spanning 4-domains A6E (*MS4A6E*, near)	11	rs10897029	59840645	T	7.317e-05<br>[1.207]	202/1850/4448 (0.1734)<br>103/870/1859 (0.19)<br>99/980/2589 (0.1606)	0.001994<br>[1.237]	97/949/2207 (0.1757)<br>56/470/978 (0.1935)<br>41/479/1229 (0.1604)	0.01262<br>[1.181]	105/901/2241 (0.1711)<br>47/400/881 (0.186)<br>58/501/1360 (0.1608)
Myosin light chain kinase (*MYLK)*	3	rs7641248	124909674	C	7.411e-05<br>[0.7978]	90/1347/5088 (0.117)<br>37/524/2280 (0.1052)<br>53/823/2808 (0.1261)	0.02176<br>[0.8309]	47/669/2544 (0.117)<br>27/275/1208 (0.1089)<br>20/394/1336 (0.124)	0.0007182<br>[0.7583]	43/678/2544 (0.117)<br>10/249/1072 (0.1011)<br>33/429/1472 (0.128)
Transcription factor B1, mitochondrial (*TFB1M)*	6	rs324356	155648017	T	7.435e-05<br>[0.8664]	1555/3236/1728 (0.4867)<br>632/1405/803 (0.4699)<br>923/1831/925 (0.4997)	0.00223<br>[0.8525]	781/1620/855 (0.4886)<br>336/757/417 (0.4732)<br>445/863/438 (0.498)	0.00969<br>[0.8767]	774/1616/873 (0.4848)<br>296/648/386 (0.4662)<br>478/968/487 (0.4977)
Kalirin, RhoGEF kinase (*KALRN)*	3	rs4678095	125439286	T	8.172e-05<br>[0.8676]	1546/3219/1758 (0.4837)<br>605/1429/806 (0.4646)<br>941/1790/952 (0.4985)	0.006406<br>[0.8695]	771/1568/919 (0.4773)<br>317/749/443 (0.4583)<br>454/819/476 (0.4937)	0.00872<br>[0.874]	775/1651/839 (0.4902)<br>288/680/363 (0.4718)<br>487/971/476 (0.4972)
SEL1L family member 3 (*SEL1L3)*	4	rs6842695	25470457	C	9.417e-05<br>[0.8053]	107/1399/5019 (0.1236)<br>42/545/2254 (0.1107)<br>65/854/2765 (0.1336)	0.02503<br>[0.8359]	54/669/2537 (0.1192)<br>20/283/1207 (0.107)<br>34/386/1330 (0.1297)	0.003529<br>[0.7969]	53/730/2482 (0.128)<br>22/262/1047 (0.115)<br>31/468/1435 (0.137)

rs7946005 and rs12166576 were excluded due to poor resolution cluster plots.
